# Complete Genome Sequences of 63 Mycobacteriophages

**DOI:** 10.1128/genomeA.00847-13

**Published:** 2013-11-27

**Authors:** Graham F. Hatfull, Science Education Alliance Phage Hunters Advancing Genomics and Evolutionary Science (SEA-PHAGES) Program, KwaZulu-Natal Research Institute for Tuberculosis and HIV (K-RITH) Mycobacterial Genetics Course, University of California—Los Angeles Research Immersion Laboratory in Virology, Phage Hunters Integrating Research and Education (PHIRE) Program

**Affiliations:** Pittsburgh Bacteriophage Institute and Department of Biological Sciences, University of Pittsburgh, Pittsburgh, Pennsylvania, USAa; Howard Hughes Medical Institute, Chevy Chase, Maryland, USAb; K-RITH, Durban, South Africac; Department of Microbiology and Immunology, Albert Einstein College of Medicine, Bronx, New York, USAd; University of California—Los Angeles Department of Microbiology, Immunology & Molecular Genetics, Los Angeles, California, USAe

## Abstract

Mycobacteriophages are viruses that infect mycobacterial hosts. The current collection of sequenced mycobacteriophages—all isolated on a single host strain, *Mycobacterium smegmatis* mc^2^155, reveals substantial genetic diversity. The complete genome sequences of 63 newly isolated mycobacteriophages expand the resolution of our understanding of phage diversity.

## GENOME ANNOUNCEMENT

The bacteriophage population encompasses amazing genetic diversity, reflecting its vast size, dynamic nature, and ancient origins (1–4). The collection of sequenced mycobacteriophage genomes offers insights into phages that infect a common host, *Mycobacterium smegmatis* mc^2^155 (5, 6), and thus are presumed to be in genetic communication with one another. Comparative genomics reveals mosaicism as the fundamental architectural feature of phage genomes, which are constructed from segments exchanged among the phage population (7, 8). Nonetheless, simple DNA comparison identifies groups of genomes more similar to one another than to other phages, and these are referred to as clusters (9). The previously reported 223 mycobacteriophages assort into a total of 15 different clusters (clusters A through O) and eight singletons that have no close relatives (5, 10–13). Several of the clusters can be divided into subclusters according to nucleotide sequence similarities (5). The total number of clusters, subclusters, and singletons is 36 (5).

Bacteriophage discovery and genomic characterization provide an effective platform for introducing students to authentic scientific research (9, 14, 15). The 63 phages described here were isolated and sequenced by undergraduate students in four programs: the Science Education Alliance Phage Hunters Advancing Genomics and Evolutionary Science (SEA-PHAGES) Program, the Phage Hunters Integrating Research and Education (PHIRE) Program, the Research Immersion Laboratory in Virology at UCLA, and the Mycobacterial Genetics Course at the University of KwaZulu-Natal, Durban, South Africa (Table 1). Students and faculty isolated, purified, named, extracted, and annotated each of the new phages.

**TABLE 1:** Data for newly sequenced mycobacteriophage genomes

Phage name	Cluster	Accession no.	Length (bp)	GC%	Institution(s)
ABCat	E	KF188414	76,131	63.0	Southern Connecticut State University^*a*^
Aeneas	A1	JQ809703	53,684	63.6	Brigham Young University^*a*^
ArcherS7	C1	KC748970	156,558	64.7	Trinity College^*a*^
Arturo	A4	JX307702	51,500	64.1	Hampden-Sydney College^*a*^
Astraea	C1	KC691257	154,872	64.7	North Carolina State University^*a*^
Astro	A8	JX015524.1	52,494	61.4	College of Charleston^*a*^
Ava3	C1	JQ911768	154,466	64.8	Calvin College^*a*^
Avani	F2	JQ809702	54,470	61.0	Gettysburg College^*a*^
Bobi	F1	KF114874	59,179	61.7	Purdue University^*a*^
Breeniome	C1	KF006817	154,434	64.8	CUNY, Queens College^*a*^
Breezona	L2	KC691254	76,652	58.9	Bucknell University^*a*^
Butters	N	KC576783	41,491	65.8	Lehigh University^*a*^
Catdawg	O	KF017002	72,108	65.4	Cabrini College^*a*^
Contagion	E	KF024732	74,533	63.1	Loyola Marymount University^*a*^
Crossroads	L2	KF024731	76,129	58.9	Teacher workshop, University of Pittsburgh^*d*^
Daenerys	F1	KF017005	58,043	61.6	University of Pittsburgh^*a*^
Dhanush	A4	KC661271	51,373	63.9	University of Alabama at Birmingham^*a*^
Dorothy	F1	JX411620	58,866	61.4	Baylor University^*a*^
Dumbo	E	KC691255	75,736	63.0	University of Maine, Fort Kent^*a*^
Dylan	O	KF024730	69,815	65.4	University of KwaZulu-Natal^*b*^
ElTiger69	A5	JX042578	51,505	59.8	University of North Texas^*a*^
Fishburne	P	KC691256	47,109	67.3	College of Charleston^*a*^
Flux	A4	JQ809701	51,370	63.9	Saint Joseph’s University^*a*^
Gizmo	C1	KC748968	157,482	64.6	Illinois Wesleyan University^*a*^
Goose	A10	JX307704	50,645	65.1	Del Mar College^*a*^
Hamulus	F1	KF024723	57,155	61.8	University of California, Santa Cruz^*a*^
HINdeR	A7	KC661275	52,617	62.8	Miami University^*a*^
ICleared	A4	JQ896627	51,440	63.9	University of Louisiana at Monroe^*a*^
Jabbawokkie	F2	KF017003	55,213	61.1	University of Pittsburgh^*a*^
Job42	F1	KC661280	59,626	61.2	Providence College^*a*^
Jobu08	A3	KC661281	50,679	64.0	Washington State University^*a*^
KayaCho	B4	KF024729	70,838	70.0	University of KwaZulu-Natal^*a*,*b*^
LittleCherry	A5	KF017001	50,690	60.9	College of William & Mary^*a*^
MacnCheese	K3	JX042579	61,567	67.3	Calvin College^*a*^
Marcell	A1	JX307705	49,186	64.0	University of Maine, Machias^*a*^
Medusa	A4	KF024733	51,384	63.9	University of Louisiana at Monroe^*a*^
Methuselah	A3	KC661272	50,891	64.2	University of Maryland, Baltimore County^*a*^
Muddy	Single	KF024728	48,228	58.8	University of KwaZulu-Natal^*b*^
Murphy	E	KC748971	76,179	62.9	Ohio State University^*a*^
Newman	B1	KC691258	68,598	66.5	University of Colorado at Boulder^*a*^
Odin	A2	KF017927	52,807	62.3	University of California, Los Angeles^*c*^
PattyP	A1	KC661273	52,057	63.6	Carthage College^*a*^
PegLeg	M	KC900379	80,955	61.5	University of California, Los Angeles^*c*^
Phaux	E	KC748969	76,479	62.9	Helena High School,^*d*^ Queensboro Community College^*a*^
Phrux	E	KC661277	74,711	63.1	Miami University^*a*^
Rebeuca	A10	JX411619	51,235	65.1	University of Texas at El Paso^*a*^
Redno2	J	KF114875	108,297	60.9	Virginia Commonwealth University^*a*^
Reprobate	B5	KF024727	70,120	68.3	University of KwaZulu-Natal^*b*^
Sabertooth	A4	JX307703	51,377	63.9	Culver-Stockton College^*a*^
SarFire	A1	KF024726	53,701	63.8	University of KwaZulu-Natal^*b*^
SDcharge11	B1	KC661274	67,702	66.5	Loyola Marymount University^*a*^
Severus	A10	KC661279	49,894	64.4	College of St. Scholastica^*a*^
ShiVal	B1	KC576784	68,355	66.5	Montclair State University^*a*^
Shrimp	C1	KF024734	155,714	64.7	Illinois Wesleyan University^*a*^
SiSi	F1	KC661278	56,279	61.5	Gonzaga University^*a*^
Taj	F1	JX121091	58,550	61.9	University of Pittsburgh^*d*^
Tiger	A5	JQ684677	50,332	60.7	Oregon State University^*a*^
Trouble	A1	KF024724	52,102	63.6	University of California, Santa Cruz^*a*^
Twister	A10	JQ512844	51,094	65.0	Virginia Commonwealth University^*a*^
Velveteen	F1	KF017004	54,314	61.5	University of Pittsburgh^*a*^
Wanda	J	KF006818	109,960	60.8	Lehigh University^*a*^
Whirlwind	L3	KF024725	76,050	59.3	University of Pittsburgh^*d*^
Winky	L2	KC661276	76,653	58.9	Saint Joseph’s University^*a*^

a SEA-PHAGES member institution.

b K-RITH Mycobacterial Genetics Course.

c UCLA Research Immersion Laboratory in Virology.

d Phage Hunters Integrating Research and Education (PHIRE) Program, University of Pittsburgh.

Phage genomic DNA was sequenced using Ion Torrent, 454 pyrosequencing, or Illumina platforms with an average redundancy of 210-fold (range, 25- to 900-fold). Reads were assembled using Newbler (version 2.6), and the assembly quality was evaluated using Consed (version 20 or newer); targeted Sanger sequencing resolved weak areas where needed. Fifty-two of the genomes have defined ends, and the termini were determined by comparison to closely related genomes, examination of read density, and where necessary, Sanger sequencing on genomic DNA. Eleven genomes assembled circularly and are presumed to have circularly permuted terminally redundant ends, and position 1 was selected at the 5′ end of the most distally closely linked gene to the terminase large subunit (1 to 5 genes upstream in the five cluster B phages). For the six cluster C phages, position 1 was chosen for consistency with Bxz1 (8).

Phage genomes were annotated using DNA Master (http://cobamide2.bio.pitt.edu/computer.htm), Glimmer 3.0 (16), GeneMark (17, 18), Aragorn (19), tRNAscan-SE (20), and Phamerator (21), and gene functions were predicted using BLAST and HHPred (22). All sequences and student-annotated genomes were reviewed and revised as necessary at the University of Pittsburgh.

Of the 63 genomes, one (Fishburne) joins a singleton to form cluster P, one (Muddy) is a new singleton, and four add new subclusters, two in A10 and one each in B5 and L5 (Table 1). The 285 sequenced mycobacteriophage genomes form a total of 39 clusters or subclusters and 8 singletons.

Further information on these phage genomes is available at http://www.phagesdb.org.

### Nucleotide sequence accession numbers.

GenBank accession numbers are shown in Table 1.
